# Exploring responsivity, sensitivity and resolution in amplitude modulated AFM: a study of global behavior and parameter influences

**DOI:** 10.1038/s41378-026-01173-9

**Published:** 2026-02-14

**Authors:** Jonathan Ehrmann, Thomas Sattel, Oliver Radler

**Affiliations:** Mechatronics Group, Technische Universität Ilmenau, Ilmenau, Germany

**Keywords:** Engineering, Sensors

## Abstract

In atomic force microscopy, sensitivity is one of the most important characteristics as in many measurement applications. Nevertheless, in literature different meanings of the terms sensitivity and resolution can be found. The same holds for the connectected quantity responsivity. Far more, there is no literature showing globally how different system and process parameters influence sensitivity. In this work we want to make a clear definition of these term in the context of AFM. Additionally, we present the global behavior of the AFM cantilever-sample system in terms of responsivity, noise, and sensitivity. An analytical model is derived that shows this system behavior. This is achieved by finding simple analytical equations for amplitude and phase as functions of the tip-sample distance assuming small amplitudes. Furthermore, the derived equations are scaled to reduce the amount of parameters and get a more generalized form. The scaled equations are analyzed to show the influence of system parameters like damping ratio, excitation frequency and sample parameters on the global system behavior. With that, parameter for best sensitivity can be found. For larger amplitudes where the analytical model is not valid, a numerical model solved with numerical continuation is used to gain further results showing the difference between non-contact and intermittent mode. For validation, we show experimental amplitude distance curves measured with a self-developed setup. This setup is a new possibility to measure amplitude distance curves in an open and flexible environment without the need of having a commercial AFM system.

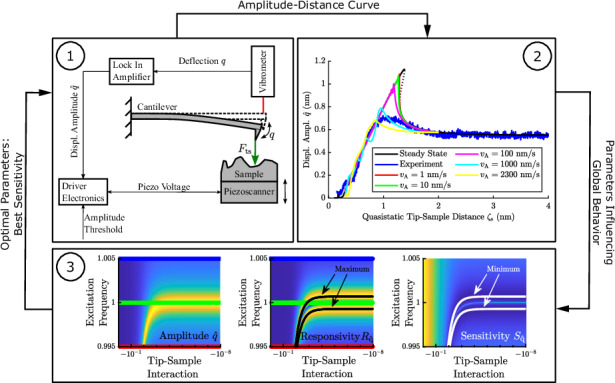

## Introduction

Atomic force microscopy (AFM) uses a cantilever-shaped MEMS probe to map the topography of a sample surface. In dynamic AFM (dAFM) with amplitude modulation, the cantilever is usually excited in the vicinity of its first bending mode eigenfrequency to oscillate over the sample. Depending on the specific mode, the cantilever touches the surface intermittently (intermittent or tapping mode) or is not in physical contact with the surface (non-contact mode). In both cases, a so-called tip-sample interaction force influences the oscillation of the cantilever with the van der Waals force as main contribution^1^. The force is nonlinear and follows^2,3^

1 $${F}_{{\rm{ts}}}\left(\zeta \right)=\frac{AR}{6}\left(\frac{1}{{\zeta }^{2}}-\frac{{a}_{0}^{6}}{30}\,\frac{1}{{\zeta }^{8}}\right),$$

with the Hamaker constant *A*, the tip radius of the cantilever *R*, the intermolecular distance of the sample molecules *a*_0_ and the tip-sample distance *ζ*. For simplification, the tip-sample interaction force can be linearized at a certain reference distance *ζ*_0_ using a Taylor series^4,5^

2 $${F}_{{\rm{ts}}}\approx {\left.{F}_{{{\rm{ts,\zeta }}}_{{\rm{0}}}}+\frac{{\rm{d}}{F}_{{\rm{ts}}}}{{\rm{d}}\zeta }\right|}_{{\zeta }_{0}}(\zeta -{\zeta }_{0})={F}_{{{\rm{ts,\zeta }}}_{{\rm{0}}}}+{k}_{{\rm{ts}}}(\zeta -{\zeta }_{0}).$$

Here, *k*_ts_(*ζ*_0_) denotes the tip-sample stiffness, meaning the slope of the tip-sample interaction force curve at a quasistatic distance *ζ*_s_ = *ζ*_0_ which leads later to an amplitude reduction due to the shift of the eigenfrequency of the cantilever^1^. It is important to note that the linearization of the tip-sample interaction force only holds for small amplitudes and is therefore not valid for intermittent mode AFM with larger amplitudes compared to non-contact AFM^6^.

As in many measuring applications, sensitivity is also in AFM one of the most important characteristics of the measuring system. However, in the context of AFM we found out, that the term sensitivity and the corresponding definition is not applied consequently and therefore investigations on sensitivity have different meanings. Most authors use the term resolution to indicate the performance of AFM^1,4,7–11^. This term is broadly understood and therefore prevents misunderstandings. Between the lines it is common knowledge that sensitivity and resolution are to be used as synonyms. Nevertheless, there are only a few works in which it is clear that resolution means the same thing as sensitivity^1,12^. Others use both terms without connecting them clearly^9^. Most of the authors use the term sensitivity more as a way to describe the dependency of measures to certain parameters changes^10,11,13^. This is later defined as responsivity.

Here, we want to apply a clear definition of sensitivity to the context of AFM. Sensitivity (and therefore resolution) is defined as the smallest detectable change of a measured quantity and is limited by noise^12^. In other words, the limitation by noise implies a signal-to-noise ratio (SNR) of one^12^. In context of amplitude modulated AFM, we see this measured quantity as the displacement amplitude of the oscillating cantilever (from now on displacement amplitude is meant with amplitude). This amplitude changes in dependence of a change of the tip-sample distance. So, the slope of the amplitude-distance curve is of interest which we call *amplitude responsivity* ${R}_{\widehat{{\rm{q}}}}$, following^12^ where responsivity is defined as the slope of a sensor output function *x*_o_ = *f*(*x*_i_) with input quantity *x*_i_ = *ζ* as the tip-sample distance and sensor output quantity ${x}_{{\rm{o}}}=\widehat{q}$ as the displacement amplitude,

3 $${R}_{\widehat{{\rm{q}}}}:=\frac{{\rm{d}}\widehat{q}}{{\rm{d}}\zeta }.$$

The same can be applied to the phase as measured quantity, leading to the *phase responsivity* *R*_*φ*_ ≔ d*φ*/d*ζ*. It is generally known that the limiting noise is of thermomechanical nature^4^. Here also, a distinction between amplitude and phase must be taken, so that amplitude noise $\delta {\widehat{q}}_{{\rm{th}}}$ and phase noise *δφ*_th_ result in^4,14^

4 $$\delta {\widehat{q}}_{{\rm{th}}}=\sqrt{4\,{k}_{B}T\,Q\,\frac{1}{{\omega }_{0}\,k}\,B},\,\,\delta {\varphi }_{{\rm{th}}}=\frac{1}{\widehat{q}}\sqrt{4\,{k}_{B}T\,Q\,\frac{1}{{\omega }_{0}\,k}\,B},$$

with the parameters of the environmental measurement conditions, *k*_B_*T* and *Q*, which are Boltzmann constant *k*_B_ and temperature *T* for the thermal influence and the quality factor of the cantilever *Q*, as well as the the cantilever parameters eigenfrequency *ω*_0_, modal stiffness *k*, then the frequency bandwidth 2*B* of the measurement and finally, the amplitude $\widehat{q}$ of the cantilever. In this context, phase means the phase lag between excitation force and cantilever displacement signal. In the most cases, the frequency bandwidth of the measurement corresponds to a band-pass filter around *ω*_0_ with the bandwidth 2*B* as for example when using a lock-in amplifier^15^.

From (4) can be seen that the amplitude noise during approach of the cantilever to the sample stays constant because neither the tip-sample distance nor the cantilever amplitude plays a role. On the other side, the phase noise changes while approaching because the amplitude $\widehat{q}$ of the cantilever is changing during approach.

Now, sensitivity is calculated by combining both responsivity and noise, resulting in the *amplitude sensitivity* ${S}_{\widehat{{\rm{q}}}}$ and *phase sensitivity* *S*_*φ*_^12^

5 $${S}_{\widehat{{\rm{q}}}}:=\frac{\delta {\widehat{q}}_{{\rm{th}}}}{{R}_{\widehat{{\rm{q}}}}},\,{S}_{\varphi }:=\frac{\delta {\varphi }_{{\rm{th}}}}{{R}_{\varphi }}.$$

It is important to notice that a low value of ${S}_{\widehat{{\rm{q}}}}$ respectively *S*_*φ*_ is desirable even if one speaks of a high sensitivity. This gets more clear if we keep in mind this definition of amplitude sensitivity is a synonym for the *spatial resolution in vertical direction*^16^. Due to this, the units of ${S}_{\widehat{{\rm{q}}}}$ and *S*_*φ*_ are nm and rad.

One existing method for applying the definition of sensitivity to AFM is to calculate the minimal detectable force gradient, which is common knowledge and was first presented by Martin et al., to the best of our knowledge. Here, the input quantity for (3) is chosen according to (2) to *x*_i_ = Δ*F*_ts_/Δ*ζ* = *k*_ts_. Depending if amplitude or phase is the measured quantity the equation is^4,14^

6 $${S}_{\widehat{{\rm{q}}},\min }=\frac{\delta {\widehat{q}}_{{\rm{th}}}}{{\rm{d}}\widehat{q}/{\rm{d}}{k}_{{\rm{ts}}}}=\frac{1}{\widehat{q}}\sqrt{27\,\frac{{k}_{{\rm{B}}}T}{Q}\,\frac{k}{{\omega }_{0}}\,B}\,{\rm{or}}\,{S}_{\varphi ,\min }=\frac{\delta {\varphi }_{{\rm{th}}}}{{\rm{d}}\varphi /{\rm{d}}{k}_{{\rm{ts}}}}=\frac{1}{\widehat{q}}\sqrt{8\,\frac{{k}_{{\rm{B}}}T}{Q}\,\frac{k}{{\omega }_{0}}\,B}$$

which is calculated from (5). The equations correspond to the best sensitivity (lowest possible sensitivity value).

With these equations, it is not only possible to see how several system parameters influence sensitivity, but it is also possible to optimize sensitivity with respect to an existing system. Because (6) is calculated for the steepest point on the resonance curve, this equation describes sensitivity only in a single operating point. Although this is the best point regarding sensitivity to operate the system, the equation is far from providing information about global system behavior in the context of responsivity, noise, and sensitivity. Also, sample parameters (see (1)) are not included from that perspective.

For this reason, in this work we explore the global behavior of amplitude and phase including responsivity and sensitivity. Global behavior means the connection between system and sample parameters with the system’s sensitivity based on the steady-state behavior. According to our knowledge, there is no work that shows these correlations at a glance. Nevertheless, this is important, especially at the beginning of AFM applications, to gain a better understanding of the global system behavior. Only a fundamental understanding of the underlying dynamics of the AFM cantilever-sample system enables an user to make a systematic and well-founded decision over the choice of system and sample parameters in order to achieve optimal conditions to enhance sensitivity. Anyway, it could help to get a first approach finding experimental parameters for first experiments without needing to have years of experimental experience.

## Results

### Analytic Approach to Sensitivity in AFM

In this section, an analytic model is derived that shows the AFM system behavior, meaning amplitude and phase of the cantilever in relation to tip-sample distance as well as responsivity and sensitivity. For that, the first bending eigenmode of the cantilever is modeled as a single degree of freedom harmonic oscillator. We use the linearized version of the tip-sample interaction force as the most simple way to include the interactions of the cantilever with the sample. This leads to analytical equations for amplitude and phase behavior which are also presented in^5^. We want to show a new approach to understand the origin of this equations.

From that base we proceed with scaling of the derived equations to reduce the amount of parameters and get a more generalized form. Then, these equations are used to calculate overview plots to show the influence of the generalized parameters on responsivity and sensitivity.

#### Amplitude and Phase Distance Behavior

To come to an analytic expression for the amplitude of a cantilever in relation to the tip-sample distance, $\widehat{q}\left(\zeta \right)$, the entire cantilever probe together with the sample must be considered as a dynamic unit consisting of several subunits. This includes the cantilever dynamics, the operating point, the change of cantilever dynamics due to the tip-sample interaction force model and the tip-sample interaction force model itself. Together, these subunits determine the behavior of the overall cantilever-sample unit. These subunits can each be expressed by individual mathematical functions $\widehat{q}(\eta ),\,\eta ({\omega }_{0,{\rm{ts}}}),\,{\omega }_{0,{\rm{ts}}}({F}_{{\rm{ts}}}),\,{F}_{{\rm{ts}}}(\zeta )$, where $\widehat{q}(\eta )$ stands for the dynamic behavior of the cantilever, meaning the amplitude as function of the excitation frequency (resonance curve). *η*(*ω*_0,ts_) is the current operating point regarding the ratio of excitation frequency to eigenfrequency, *ω*_0,ts_(*F*_ts_) the angular eigenfrequency dependent on the tip-sample interaction force (which already implies the linearization of *F*_ts_, (2)) and *F*_ts_(*ζ*_s_) the tip-sample interaction force model itself. Note, that *ζ*_s_ stands for the quasistatic tip-sample distance which neglects the oscillation of the cantilever and only take into account the quasistatic deflection of the cantilever due to the tip-sample interaction force. This is a valid assumption for small oscillation amplitudes^5,6^. Details of the kinematics are described in Materials and Methods. By chaining these functions we get an expression for the cantilever amplitude $\widehat{q}$ as function of the quasistatic tip-sample distance *ζ*_s_

7 $$\widehat{q}({\zeta }_{{\rm{s}}})=\widehat{q}(\eta )\circ \,\eta ({\omega }_{0,{\rm{ts}}})\circ \,{\omega }_{0,{\rm{ts}}}({F}_{{\rm{ts}}})\circ {F}_{{\rm{ts}}}({\zeta }_{{\rm{s}}}).$$

As explained, linearization of *F*_ts_ leads to

8 $$\widehat{q}({\zeta }_{{\rm{s}}})=\mathop{\underbrace{\widehat{q}(\eta )}}\limits_{(9)}\circ \,\mathop{\underbrace{\eta ({\omega }_{0,\mathrm{ts}})\circ {\omega }_{0,\mathrm{ts}}({k}_{\mathrm{ts}})}}\limits_{(11)}\circ \mathop{\underbrace{{k}_{\mathrm{ts}}({\zeta }_{{\rm{s}}})}}\limits_{(12)}.$$

Now, the chain links in (8) are considered individually. $\widehat{q}(\eta )$ corresponds to the magnification function of a harmonic oscillator whose excitation amplitude ${\widehat{w}}_{{\rm{exc}}}$ is selected that the cantilever amplitude in the resonance maximum ${\eta }_{{\rm{R}}}=\sqrt{1-2{D}^{2}}$ is $\widehat{q}={\widehat{q}}_{0}$ which results in

9 $$\widehat{q}(\eta )=\frac{{\widehat{w}}_{{\rm{exc}}}}{\sqrt{{\left(1-{\eta }^{2}\right)}^{2}+{\left(2D\eta \right)}^{2}}}=\frac{2D\sqrt{1-{D}^{2}}}{\sqrt{{\left(1-{\eta }^{2}\right)}^{2}+{\left(2D\eta \right)}^{2}}}\,{\widehat{q}}_{0}.$$

Here, *D* is the damping ratio and $Q=\frac{1}{2D\sqrt{1-{D}^{2}}}$. With small damping *D* < 0.1 this results in

10 $$Q\approx \frac{1}{2D}.$$

The ratio of the fixed excitation frequency Ω to the eigenfrequency of the combined cantilever-sample system *ω*_0,ts_^1^ is represented by

11 $$\eta ({\omega }_{0,{\rm{ts}}})=\frac{\Omega }{{\omega }_{0,{\rm{ts}}}}\,{\rm{with}}\,{\omega }_{0,{\rm{ts}}}=\sqrt{1+\frac{{k}_{{\rm{ts}}}}{k}}\,{\omega }_{0}.$$

Last, the tip-sample stiffness (see (2)) as function of the quasistatic tip-sample distance *ζ*_s_ which is responsible for the shift of the eigenfrequency of the cantilever

12 $${k}_{{\rm{ts}}}({\zeta }_{{\rm{s}}})=\frac{AR}{3}\left(-\frac{1}{{\zeta }_{{\rm{s}}}^{3}}+\frac{2\,{a}_{0}^{6}}{15}\,\frac{1}{{\zeta }_{{\rm{s}}}^{9}}\right).$$

Chaining all subfunctions and inserting them into each other in (8) provides an analytical expression for the amplitude-distance curve

13 $$\widehat{q}({\zeta }_{{\rm{s}}})=\frac{2D\sqrt{1-{D}^{2}}}{\sqrt{{\left(1-\frac{{\eta }_{0}^{2}}{1-\frac{AR}{3{\zeta }_{{\rm{s}}}^{3}k}+\frac{2AR{a}_{0}^{6}}{45{\zeta }_{{\rm{s}}}^{9}k}}\right)}^{2}+{\left(\frac{2D{\eta }_{0}}{1-\frac{AR}{3{\zeta }_{{\rm{s}}}^{3}k}+\frac{2AR{a}_{0}^{6}}{45{\zeta }_{{\rm{s}}}^{9}k}}\right)}^{2}}}\,{\widehat{q}}_{0},\,\,\,\,\,\mathrm{with}\,\,\,\,\,{\eta }_{0}=\Omega /{\omega }_{0},$$

where *η*_0_ is the (fixed) frequency ratio before approaching the sample (frequency setpoint).

The same can be done for the phase distance curve. The procedure is the same as with the amplitude distance function. We also start with the chained functions of the subunits

14 $$\varphi ({\zeta }_{{\rm{s}}})=\varphi (\eta )\circ \eta ({\omega }_{0,{\rm{ts}}})\circ {\omega }_{0,{\rm{ts}}}({F}_{{\rm{ts}}})\circ {F}_{{\rm{ts}}}({\zeta }_{{\rm{s}}}),$$

where

15 $$\varphi (\eta )=\arctan \frac{-2D\eta }{1-{\eta }^{2}}$$

is the phase of the harmonic oscillator. From this, we get the phase distance curve of the cantilever

16 $$\varphi ({\zeta }_{{\rm{s}}})=\arctan \frac{-2D\eta }{\left(1-\frac{{\eta }_{0}^{2}}{1-\frac{AR}{3{\zeta }_{{\rm{s}}}^{3}k}+\frac{2AR\,{a}_{0}^{6}}{45\,{\zeta }_{{\rm{s}}}^{9}k}}\right)\sqrt{1-\frac{AR}{3{\zeta }_{{\rm{s}}}^{3}k}+\frac{2AR\,{a}_{0}^{6}}{45\,{\zeta }_{{\rm{s}}}^{9}k}}}.$$

#### Validation of Analytical and Numerical Model Using Experimental Data

**Fig. 1:**
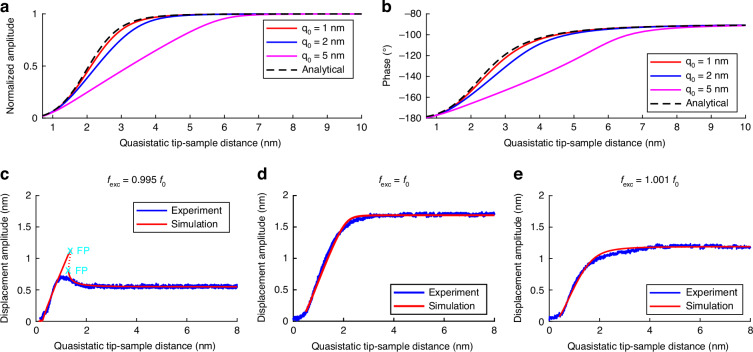
Model validation. **a, b** Validation of analytical equations (13), (16) for amplitude-/phase-distance curves. For ${\widehat{q}}_{0}\le 1$ nm, the equations are considered as validated. **c–e** Experimental (blue) and numerical (red) results show agreement

First, to validate the analytical equations derived before, a comparison is done. For this, the Continuation Core Toolbox^17^ is used to numerically calculate amplitude-/phase-distance curves for different free amplitudes of ${\widehat{q}}_{0}=\{1,\,2,\,5\}$ nm. Free amplitude means the amplitude that results when the cantilever is excited at eigenfrequency and the approach distance to the sample is large enough that the tip-sample interaction forces can be neglected. In contrast to Eqs. (13) and (16), the numerical model considers the nonlinear tip-sample interaction force (1) as well as the actual tip-sample distance *ζ* at every computation step without averaging. The detailed procedure is described in Materials and Methods as well as the used parameters. Figure 1a–b shows the normalized amplitude-distance and phase-distance curves together with (13) and (16). The normalized analytical curves are identical for different free amplitudes ${\widehat{q}}_{0}$ because of the linearity of the equations. Until a free amplitude of 1 nm, we consider the equations as valid, based on the strong deviations for larger amplitudes. This corresponds to the literature, where oscillation amplitudes of a few nanometers mark the absolute limit of the simple analytical model^1^. The numerical model is further validated using experimental data. The experimental procedure is described in Materials and Methods. In Fig. 1c–e the experimental curves are shown together with the numerical results for different excitation frequencies ${f}_{{\rm{exc}}}=\left\{0.995,\,1,\,1.001\right\}{f}_{0}$. For comparison, the 3dB frequency bandwidth of the resonance is *B* = 228 Hz which corresponds to an excitation frequency *f*_exc_ = 0.999*f*_0_ left of the resonance. The experimental curves show agreement with the numerical results, where the only difference is that the increase of the tip amplitude in Fig. 1c is in the experiment lower than simulated. We see the reason in the fact that the experimental curve was gained by continuously decreasing the tip-sample distance while measuring the amplitude $\widehat{q}$. We call this kind of approach a *continuous approach* where the approach velocity *v*_a_ ≠ 0. In contrast, the numerical results represent a so-called *steady-state approach* with *v*_a_ = 0. Therefore, the velocity of the z-scanner could have prevented a full increase of the amplitude to the value of the numerical simulation. Furthermore, the numerical model only includes the described tip-sample interaction model (see (1)). In addition to that, there could be some more effects that influence the amplitude of the cantilever, for example, dissipative forces^1^.

**Fig. 2:**
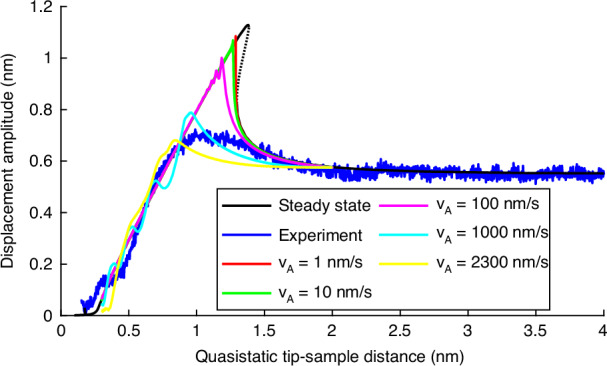
Comparison of the computed steady-state solution (*v*_a_ = 0) and the continuous approaching experimental data (*v*_a_ = 2300 nm/s) from Fig. 9c with numerically computed continuous approaches for different approach velocities *v*_a_. Low approach velocities correspond to the steady-state solution where high approach velocities fit to the experimental curve

For better understanding, this deviation of simulation and experiment in Fig. 1c should be further investigated by setting up another type of simulation. Now, the amplitude-distance curve is computed during a continuous approach of the cantilever to the sample for different approach velocities *v*_a_ ≠ 0, in contrast to the steady-state solutions used in the rest of this work. A detailed description of the simulation is given in Materials and Methods. Figure 2 shows a comparison of the computed steady-state approach and the experimental continuous approach (as already shown in Fig. 1c), with the computed continuous approach for different approach velocities *v*_a_. It can be seen that for very slow approaches (*v*_a_ = 1 nm/s and *v*_a_ = 10 nm/s) the steady-state solution and the continuous approach solution are identical except for the small region that is not reached because of the jump at the fold point (FP). The latter is a completely expected behavior and a general phenomenon in dynamics. As the approach velocity increases, the deviation to the steady-state solution increases as well, meaning that the jump of the amplitude happens at smaller tip-sample distances and is therefore not so high. Additionally, the amplitude oscillation after the jump gets stronger the faster the approach is. For the highest values of *v*_a_, the computed curves get closer to the experimental curve. This is remarkable because the approach velocity of the experiment *v*_a_ = 2300 nm/s is similar to that of the yellow curve. The missing amplitude oscillations in the experimental curve could be explained by additional interaction forces, as stated before. With that analysis, we see that the observed deviation of the experiment to the simulation in Fig. 1c can indeed be explained with the approach velocity *v*_a_ ≠ 0 in the experiment compared to a computed steady-state solution.

To summarize the model validation, the derived analytical model ((13), (16)) holds for small amplitudes, namely ${\widehat{q}}_{0}\le 1$ nm. In contrast, the numerical model has no limitations regarding the value of the free amplitude ${\widehat{q}}_{0}$. Nevertheless, both models have not implemented the approach velocity because they calculate steady-state responses. Therefore, they are only valid for very small approach velocities (Fig. 2). Lastly, for cases of additional deviations between simulation and experimental results larger than those presented here, the model should be expanded to include further interaction force models, such as dissipative forces or those occurring during operation in liquids, in order to resolve these deviations.

#### Reduction of Model Parameters

After validating the developed models, the goal is to get a more global overview how parameters influence the amplitude and phase behavior of the cantilever in interaction with the sample. Therefore, the derived Eqs. (13) and (16) are scaled. This is done as preliminary step for the sensitivity considerations. We introduce a dimensionless tip-sample stiffness

17 $${\kappa }_{{\rm{ts}}}({\zeta }_{{\rm{s}}})=\frac{{k}_{{\rm{ts}}}({\zeta }_{{\rm{s}}})}{k}$$

which allows us to investigate Eqs. (13) and (16) in a more abstract manner. Inserting subsequently *κ*_ts_ from (17) and (12) into (13) yields

18 $$\widehat{q}({\kappa }_{{\rm{ts}}})=(1+{\kappa }_{{\rm{ts}}})\frac{2D\sqrt{1-{D}^{2}}}{\sqrt{{\left(1+{\kappa }_{{\rm{ts}}}-{\eta }_{0}^{2}\right)}^{2}+{\left(2D{\eta }_{0}\right)}^{2}}}\,{\widehat{q}}_{0},\,\varphi ({\kappa }_{{\rm{ts}}})=\arctan \frac{-2D{\eta }_{0}\sqrt{1+{\kappa }_{{\rm{ts}}}}}{1+{\kappa }_{{\rm{ts}}}-{\eta }_{0}^{2}}.$$

We have now a formulation that includes cantilever and sample characteristics as well as the tip-sample distance *ζ*_s_ in one parameter *κ*_ts_. This simplification makes it possible to analyze the influence of the cantilever (*k*), the tip-sample interaction (*A*, *R*, *a*_0_) and the tip-sample distance *ζ*_s_ with just one parameter. It facilitates the investigation of the qualitative behaviour of the system. The parameter *κ*_ts_ acts as a nondimensional frequency shift of the cantilever’s eigenfrequency as we see later.

As mentioned in Introduction, the responsivity of the cantilever is defined as the slope of the amplitude/phase over tip-sample distance. Because of the reduction of model parameters (18), the responsivity is now defined as the slope of amplitude/phase over dimensionless tip-sample stiffness by deriving (18) to *κ*_ts_,

19 $${R}_{\widehat{{\rm{q}}}}:=\left|\frac{{\rm{d}}\widehat{q}}{{\rm{d}}{\kappa }_{{\rm{ts}}}}\right|,\,{R}_{\varphi }:=\left|\frac{{\rm{d}}\varphi }{{\rm{d}}{\kappa }_{{\rm{ts}}}}\right|$$

which results in the *amplitude responsivity*

20 $${R}_{\widehat{{\rm{q}}}}=\left|\frac{{\rm{d}}\widehat{q}}{{\rm{d}}{\kappa }_{{\rm{ts}}}}\right|=\left|\frac{2D\sqrt{1-{D}^{2}}}{\sqrt{{\left(1+{\kappa }_{{\rm{ts}}}-{\eta }_{0}^{2}\right)}^{2}+{\left(2D{\eta }_{0}\right)}^{2}}}-\frac{\left(1+{\kappa }_{{\rm{ts}}}\right)\left(1+{\kappa }_{{\rm{ts}}}-{\eta }_{0}^{2}\right)2D\sqrt{1-{D}^{2}}}{{\left({\left(1+{\kappa }_{{\rm{ts}}}-{\eta }_{0}^{2}\right)}^{2}+{\left(2D{\eta }_{0}\right)}^{2}\right)}^{3/2}}\right|\,{\widehat{q}}_{0}$$

and *phase responsivity*

21 $${R}_{\varphi }=\left|\frac{{\rm{d}}\varphi }{{\rm{d}}{\kappa }_{{\rm{ts}}}}\right|=\left|\frac{D{\eta }_{0}\left(1+{\kappa }_{{\rm{ts}}}+{\eta }_{0}^{2}\right)}{\sqrt{1+{\kappa }_{{\rm{ts}}}}\left({\eta }_{0}^{4}+2\left(2{D}^{2}-1\right)\left(1+{\kappa }_{{\rm{ts}}}\right){\eta }_{0}^{2}+{\left(1+{\kappa }_{{\rm{ts}}}\right)}^{2}\right)}\right|.$$

The sensitivity of a system is not only determined by the responsivity but also limited by noise which is of thermomechanical nature in this case. Because the thermomechanical noise is not directly dependent on the tip-sample interaction, (4) cannot be scaled with the tip-sample stiffness *k*_ts_. Looking at the equation, it is clear that the amplitude noise is constant over the tip-sample interaction and the phase noise is determined by the current amplitude of the cantilever. So, for later including the thermomechanical noise in the color plots in Fig. 4 the stiffness of the cantilever *k* needs to be set, see Table 1 (*Cantilever 1*). Additionally, (4) is transformed to get nondimensional frequencies,

**Fig. 3:**
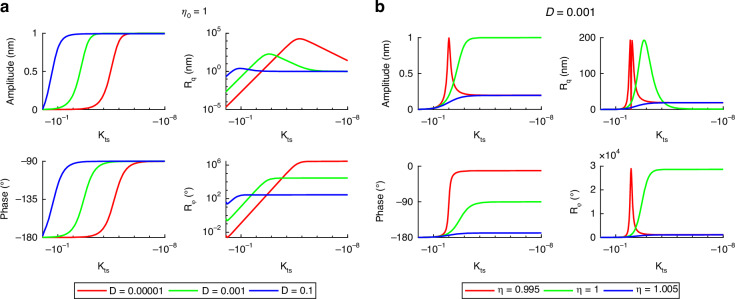
Amplitude and phase distance curves as well as amplitude and phase responsivity following the analytical equations. The curves show dependence on **a** damping ratio *D* and **b** excitation frequency *η*_0_. Excitation frequency in **a** is *η*_0_ = 1. Damping ratio in **b** is *D* = 0.001

22 $$\delta {\widehat{q}}_{{\rm{th}}}=\sqrt{4\,{k}_{B}T\,Q\,\frac{1}{{\omega }_{0}\,k}\,B}\,\sqrt{\frac{\Omega }{\Omega }}=\sqrt{4\,{k}_{B}T\,Q\,\frac{{\eta }_{0}}{k\,{\eta }_{{\rm{B}}}}},\,{\rm{with}}\,{\eta }_{{\rm{B}}}=\frac{\Omega }{B}.$$

*k*_B_, *T*, *η*_B_ need to be defined as a constant. This is no problem except for the nondimensional measurement bandwidth *η*_B_ because this is a process parameter. This is neglected for this investigation and typical values are set for *η*_B_, *k*_B_, *T*, see Table 1. Analogous, the same transformation is done for the phase noise. Now, using the sensitivity definitions (5), the thermomechanical noise in (22) together with the amplitude and phase responsivities in Eqs. (20), (21) and the damping-quality factor relation (10) results in the sensitivities ${S}_{\widehat{{\rm{q}}}}$ and *S*_*φ*_.

#### Global steady-state system behavior

Global system behavior concretely means exploring the system characteristics, amplitude and phase $\widehat{q},\,\varphi$, responsivities ${R}_{\widehat{{\rm{q}}}},\,{R}_{\varphi }$ and sensitivities ${S}_{\widehat{{\rm{q}}}},\,{S}_{\varphi }$ of the cantilever with respect to variations in the process parameters (*η*_0_, *κ*_ts_) and the environmental parameter *D*. These variations are for given and fixed cantilever parameters (*k*, *ω*_0_), measurement bandwidth parameter *η*_B_, environmental parameter temperature *T* and tip-sample interaction parameters (*A*, *R*, *a*_0_, *ζ*_s_). Thus, the analysis is reduced to

23 $$X({\eta }_{0},\,{\kappa }_{{\rm{ts}}},\,D)\,{\rm{with}}\,X\in \{\widehat{q},\,\varphi ,\,R,\,S\}.$$

As starting point, the derived equations (18), (20) and (21) are shown in Fig. 3 for different values of damping ratio *D* on the left and excitation frequency *η*_0_ on the right. On the left side, the excitation frequency is fixed to *η*_0_ = 1 and on the right side, the damping ratio is fixed to *D* = 0.001. It is important to note that the axes for *κ*_ts_ and amplitude/phase responsivity have a logarithmic scale. So, the different amplitude and phase curves in a) do not have the identical slope, even if it looks like. Also in a), amplitude and phase curves seem to have a zero gradient for *κ*_ts_ → 0. This is also not the case. Instead, they all have different values depending on the damping ratio. This can be explained, looking at (18). In this equation, the amplitude/phase curves were derived as an magnification function that is influenced by the dimensionless tip-sample stiffness *κ*_ts_ in a way that the eigenfrequency is shifted by *κ*_ts_. Additionally, depending on the damping ratio these curves are shifted along the *κ*_ts_-axis. Therefore, the slope at *κ*_ts_ = 0 is not zero for *D* ≠ 0. The most important information is that the maximum responsivity in amplitude and phase depends on the damping ratio *D*, meaning that lower damping ratios lead to higher responsivities. This fits to the state of the art, looking to (6), where the minimal detectable force gradient agrees with that statement.

**Fig. 4:**
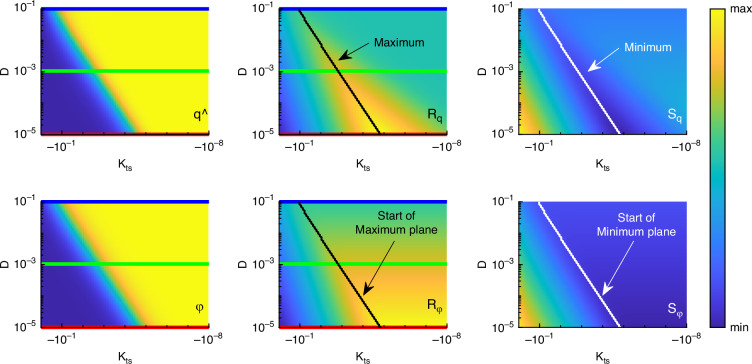
Overview of the behavior of AFM cantilever dynamics. Column-wise showing amplitude and phase, amplitude and phase responsivity and amplitude and phase sensitivity for a variation of damping ratio and nondimensional tip-sample stiffness. Blue, green and red lines mark the parameters of Fig. 3 Black lines indicate maximum responsivity and white lines indicate minimum sensitivity

In contrast to a varying damping ratio where a change of *D* does not change the qualitative shape of amplitude/phase behavior, the excitation frequency *η*_0_ has a significant influence. In Fig. 3b this influence is illustrated. Having an excitation frequency below resonance, *η*_0_ < 1, first leads to an increase in amplitude when increasing the tip-sample interaction *κ*_ts_ before the amplitude goes down to zero. Looking to the phase, its range increases so that it starts from higher values than − 90^∘^ compared to resonance case with *η*_0_ = 1. For *η*_0_ > 1 the maximum amplitude is below the free amplitude. The same holds for the phase. This leads to a reduced maximum of responsivity. The reason for this observation is quite obvious and lies in the shift of the eigenfrequency and with that the resonance curve to lower frequencies.

To gain a more global overview, Fig. 4 shows the quantities $\widehat{q},\,\varphi ,\,{R}_{\widehat{{\rm{q}}}},\,{R}_{\varphi },\,{S}_{\widehat{{\rm{q}}}},\,{S}_{\varphi }$ in dependence of *κ*_ts_ with a new dimension showing the damping ratio *D* while keeping *η*_0_ = 1 constant. With that it is possible to see how damping influences the cantilever-sample dynamics more clearly. The blue, green and red line indicate the parameters from Fig. 3, so that one curve in Fig. 3 equals one row in Fig. 4. Additionally, in the top middle plot the black line marks the maximum values of the amplitude responsivity regarding the parameter *D*. This line can also be calculated by deriving (20) to *κ*_ts_ and solve for the roots after *D*. Connecting to Fig. 3, this means connecting all the maxima with a diagonal line. The value of *R*_q_ along the line is not constant, meaning that the maximum line has a slope unequal to zero and the maximum value rises with decreasing damping ratio *D*. This leads to the imagination that the regions of high responsivity in the vicinity of the maximum line (yellow/orange) have variable broadness which is not the case. Instead, the responsivity area consists of two planes intersecting in the maximum line. The phase responsivity has no dedicated maxima but more a maximum plane. The beginning of this plane is defined by the same black line as in the amplitude responsivity plot.

In the right column of Fig. 4 the minimum values of the sensitivity are marked with a white line. Comparing this to the responsivities one can see that the black and white line correspond in the case of amplitude while in case of phase the maximum of responsivity is more a plane. This plane is also there for the phase sensitivity, forming a minimum plane. The influence of the dependence of the phase noise on the amplitude (4) only leads to steeper slope of the phase sensitivity left of the minimum plane.

The notable distinction between the amplitude and phase behavior lies in the presence of a minimum plane, as opposed to a minimum line, which affords a larger parameter range wherein optimal sensitivity is achieved (refer to the size of the blue regions in plots ${S}_{\widehat{{\rm{q}}}}$ and *S*_*φ*_). Consequently, employing the phase signal rather than the amplitude for measurement may facilitate a more stable operation of the system. Changing the specific value of the cantilever stiffness *k* just leads to a shift of the noise level in the system and a shift of the distance *ζ*_s_, where the minimum amplitude sensitivity occurs, and is therefore not to important, the qualitative shape of Fig. 4 is not influenced. This is the reason why we just show a qualitative colorbar instead of values.

**Fig. 5:**
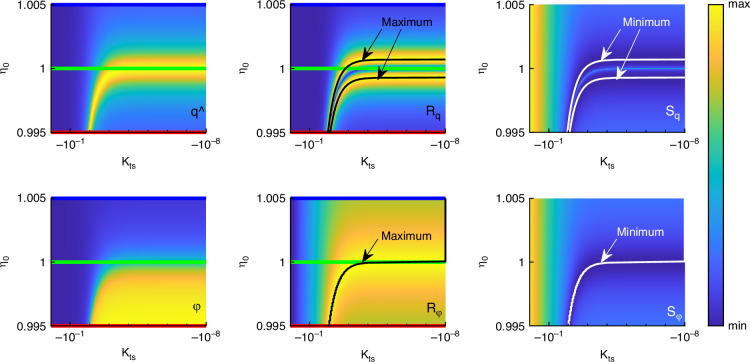
Overview of the behavior of AFM cantilever dynamics. Column-wise showing amplitude and phase, amplitude and phase responsivity and amplitude and phase sensitivity for a variation of excitation frequency and nondimensional tip-sample stiffness. Blue, green and red lines mark the parameters of Fig. 3 Black lines indicate maximum responsivity and white lines indicate minimum sensitivity

Doing the same for a variation of the excitation frequency *η*_0_ while setting *D* = 0.001 emphasizes the influence of that parameter on the qualitative shape of the curves as shown in Fig. 5. This was already indicated in Fig. 3b. Again, the blue, green and red line represent the parameters in Fig. 3b. The difference regarding responsivity in contrast to the variation of the damping ratio is that now the amplitude responsivity has two maximum lines which leads to also two minimum lines in amplitude sensitivity. The phase responsivity has a dedicated maximum line instead of a plane and with that a minimum line in phase sensitivity. The lines with maximum responsivity can also be calculated analytically as described before.

Because of the linearization of *F*_ts_, according to (2), that build the base of the used equations the results obtained in this section are just for small amplitudes applicable. Therefore, in the next section numerical simulations are done to see how the results develop for larger amplitudes leaving the pure non-contact mode and going over to intermittent mode.

### Numerical Approach to Sensitivity in AFM

**Fig. 6:**
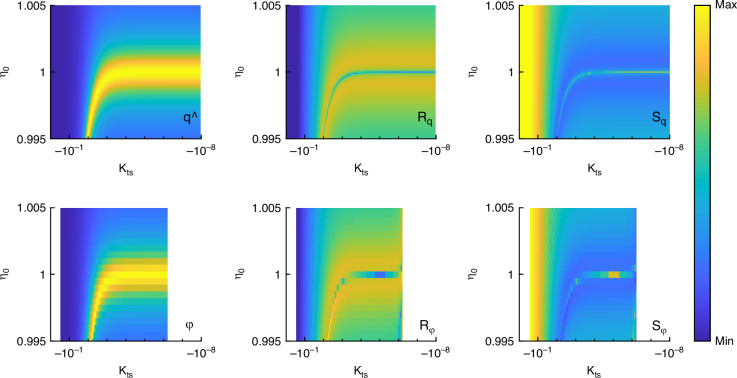
Comparison of analytical and numerical results for a free amplitude of 1 nm. Columnwise amplitude $\widehat{q}$, amplitude responsivity ${R}_{\widehat{q}}$, amplitude sensitivity ${S}_{\widehat{q}}$ while first row shows analytical, second row numerical data

The numerical simulations are carried out as described in Materials and Methods. They rely on the nonlinear tip-sample interaction force from Eq. (1). To start, in Fig. 6 the analytical results gained before are compared with corresponding numerical simulations using the same parameters. The first row shows the analytical data and the second row the numerical data which show agreement. Columnwise, the amplitude, amplitude responsivity and amplitude sensitivity is shown. The agreement also holds for the corresponding phase data and variation of the damping ratio which is not shown here. Observe that the narrower *κ*_ts_ range in the numerical outcomes can be attributed to the computation utilizing actual parameters and the derivation of *κ*_ts_ during post-processing. Specifically, the lower boundary of this numerical *κ*_ts_ interval indicates contact with the sample, for example.

Up to here, the investigation considers linear system behavior. Now it is of interest how the system behaves for amplitudes greater than ${\widehat{q}}_{0}=1$ nm where the nonlinear regime is entered. This cannot be calculated analytically anymore as proved in Fig. 1 but can be realized with the numeric simulation.

**Fig. 7:**
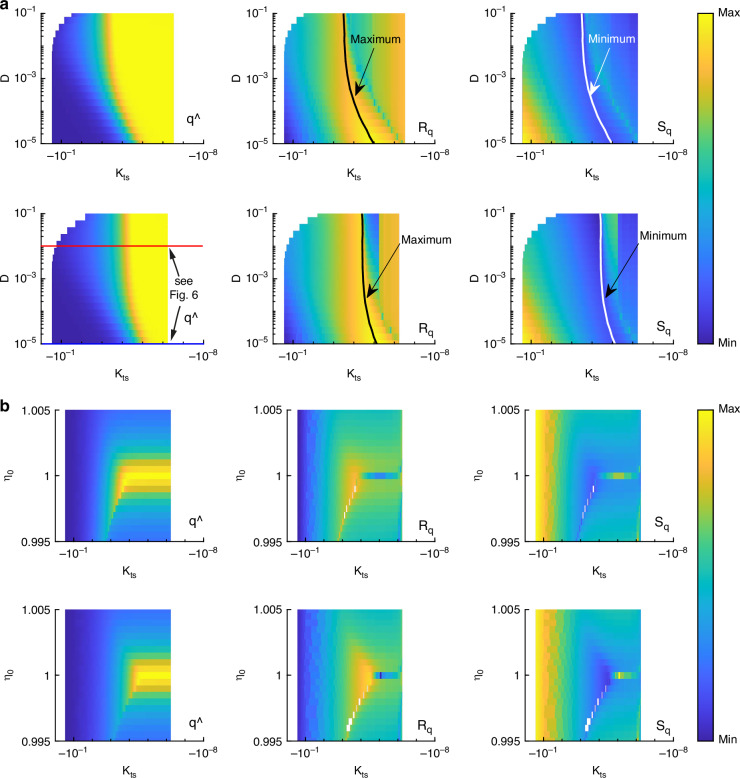
Results for larger amplitudes. **a** Numerical results for a variation of damping ratio and a free amplitude of ${\widehat{q}}_{0}=5\,{\rm{nm}}$ (first row) and 10 nm (second row). The bend of the maximum/minimum line marks the change of operation mode to intermittend mode. **b** Numerical results for a variation of excitation frequency and a free amplitude of 5 nm (first row) and 10 nm (second row). It can be seen that the character of the solution plots changes for large amplitudes

**Fig. 8:**
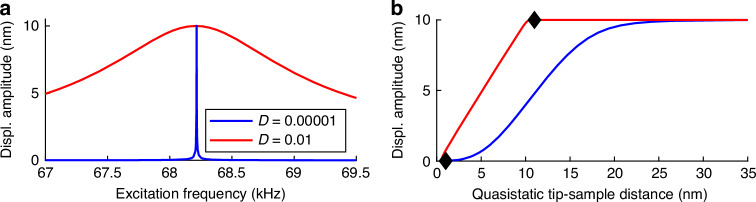
Effect of damping ratio. Frequency response plot **a** and amplitude distance plot **b** for different damping ratios with a free amplitude of ${\widehat{q}}_{0}=10$ nm. The diamond marks the begin of intermittent regime (actual tip-sample distance *ζ* ≤ 1 nm^16^

First, a variation of the damping ratio is analyzed with free amplitudes of 5 nm and 10 nm shown in Fig. 7a. Note that the yellow regions in the ${R}_{\widehat{{\rm{q}}}}$ plots for the highest values of *κ*_ts_ must be blue (${R}_{\widehat{{\rm{q}}}}\to 0$). This is due to numerical artifacts and neglected further. Increasing the free amplitude leads to a bend of the maximum/minimum line compared to Fig. 4. The maximum/minimum lines from that plot is repeated in Fig. 7a with dashed lines. The explanation for the bend lies in the change in operation regime along the *D*–axis. Along the new maximum/minimum lines, the operation regime changes continuously from non-contact to intermittent, and the cantilever experiences more and more short range forces. The explanation for the fact that the damping ratio determines the operation mode (non-contact or intermittent) can be shown by looking at exemplary frequency response and amplitude-distance curves in Fig. 8. Here it gets clear that a high damping ratio leads to less amplitude reduction for the same tip-sample interaction compared to a low damping ratio. Therefore, the actual tip-sample distance *ζ* is smaller for a wide range of *ζ*_s_ (see Materials and Methods for the kinematic relations). For *ζ*≤1 nm the operation regime can be seen as intermittent^16^. Coming from high values of *ζ*_s_ in Fig. 8b, the beginning of this regime is marked with a diamond in the plot. The red curve (high damping ratio) has the beginning of intermittent mode already at the beginning of amplitude reduction, the blue curve (low damping) almost at the end (pure non-contact mode). The red and blue curve are also marked in Fig. 7a ($\widehat{q}$, second row). The larger the free amplitude the larger gets the region of intermittent mode and non-contact mode is not possible anymore. This can be seen comparing the first and second row in Fig. 7a.

In Fig. 7b, the same analysis is done for a variation of the excitation frequency. It gets clear, that the regions of best sensitivity for *η*_0_ < 1 that are present for small amplitudes (see Fig. 5) vanish with larger free amplitude, marks by white pixels in the plots. The reason for this lies in the fold bifurcations that occur for larger free amplitudes, whereby the nonlinear behavior of the system appears clearly. This is exemplary shown in Fig. 1c (cyan crosses). The measured curves cannot follow the instable solution branch. Therefore, for calculating the threedimensional plots, the calculated solutions (red lines in Fig. 1) are cut at the fold point to get a more realistic behavior as in the measured curves. In the end, this leads to a lower but more realistic sensitivity at these points compared to full consideration of the theoretical curves. By this phenomenon, the character of the threedimensional plots changes for larger free amplitudes.

## Discussion

As said in the beginning, the term sensitivity is often mixed up in literature why we gave a comprehensive exploration of responsivity and sensitivity in the context of AFM. Based on this an analytical and linearized model for non-contact regime is derived to calculate amplitude and phase distance curves leading to a new way of exploring the global behavior of the AFM cantilever-sample system regarding sensitivity. The analytical model is confirmed using numerical simulations showing that the model is valid until a free amplitude of 1 nm which corresponds to non-contact mode. The numerical model is validated by experimental amplitude distance curves. The developed experimental setup used for Fig. 1 described in detail in Materials and Methods is a new possibility to measure amplitude distance curves in an open and flexible environment without the need of having a commercial AFM system. With the numerical model the global behavior for larger free amplitudes (intermittent mode) is further calculated. With that we can see differences between non-contact and intermittent operation regimes. In the case of a variation of the damping ratio *D* (Fig. 7a), the differences between the regimes only lay in the distortion or bending of the resulting plots. The character, meaning the existing of the minimum lines of sensitivity, stays the same. For a variation of the excitation frequency *η* (Fig. 7b) the qualitative character of the results changes in a way that the former regions of high sensitivity that are existing in non-contact regime vanish when the regime is changed to intermittent contact.

With this work, we want to provide a basis for well-founded decisions regarding first parameter sets for experiments. For example, having a specific sample in a specific environment (meaning damping ratio) to investigate, the shown results help to determine specific process parameters like cantilever stiffness and excitation frequency to get best sensitivity. This can be done by calculating an individual color plot as shown above, corresponding to a specific damping ratio. The plot then shows for which values of *κ*_ts_ and *η* the sensitivity is best. On that base, cantilever parameters as the stiffness can be chosen. In addition, it should be possible to calculate further results for specific cases in order to get an overview of the system behavior. Nevertheless, note that the chosen parameters and the operation mode are also based on other arguments such as, for example, the resulting forces that the sample experiences due to tip contact. Here, we only consider the dynamics of the cantilever-sample system. In any way, the presented results can be used to optimize an individual given system regarding sensitivity.

## Materials and Methods

### Kinematics of Cantilever-Sample System

**Fig. 9:**
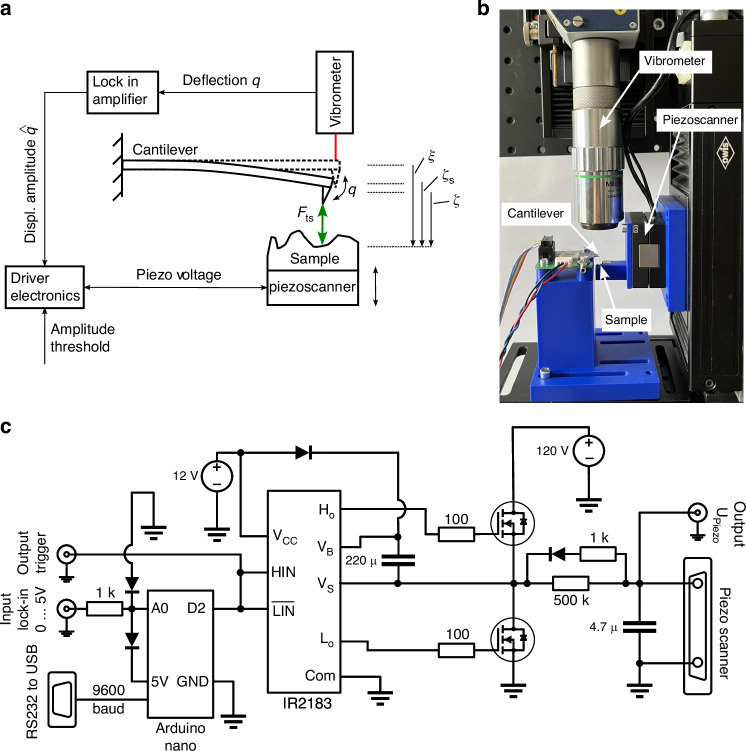
Experimental setup for measuring amplitude-distance curves. **a** Schematic setup for measuring amplitude-distance curves. The cantilever amplitude is measured by a laser vibrometer and lock in amplifier. Self-build driver electronics charge and discharge the piezo scanner depending on the current cantilever amplitude. **b** Part of measurement setup showing vibrometer, piezoscanner, active cantilever and sample. **c** Schematic of the developed analog driver electronics

To make a clear definition of the used coordinates, Fig. 9a visualizes the kinematics of the cantilever-sample system. The approach distance *ξ* is the distance to which the cantilever is approached to the sample. At this distance, the tip-sample interaction force is already noticeable. Due to this, a quasistatic tip-sample distance *ζ*_s_ follows out of the quasistatic displacement of the cantilever. If the cantilever starts oscillating, *ζ*_s_ marks the equilibrium distance to the sample. The actual tip-sample distance *ζ* considers the actual displacement of the cantilever *q* due to its oscillation and can be calculated with *ζ* = *ζ*_s_ − *q*.

As written before, the analytical equations (13) and (16) use the quasistatic tip-sample distance *ζ*_s_ and the numerical model considers the actual tip-sample distance *ζ*.

### Numerical Simulation

Numerical simulations are carried out in Matlab using time integration solvers and numerical continuation (Continuation Core Toolbox^17^). The cantilever probe is modeled by the equation of motion as a single degree of freedom lumped mass oscillator

24 $$\ddot{q}+2D{\omega }_{0}\mathop{q}\limits^{^\circ }+{\omega }_{0}^{2}q=\frac{\widehat{F}}{m}\cos \Omega t+\frac{{F}_{{\rm{ts}}}}{m}.$$

The tip-sample interaction force is calculated using the actual tip-sample distance *ζ* at each computation step, see Fig. 9a. (24) is scaled for better numerical results using *τ* = *ω*_0_*t* where *ω*_0_ is the free (without tip-sample interaction) eigenfrequency of the cantilever. All parameters with length units are scaled to *q*_ref_ = 1 nm. With that, (24) transforms to

25 $${\bar{q}}^{{\prime\prime} }+2D{\bar{q}}^{{\prime} }+\bar{q}=\frac{\widehat{F}}{m{\omega }_{0}^{2}{q}_{{\rm{ref}}}}\cos {\eta }_{0}\tau +\frac{{F}_{{\rm{ts}}}(\zeta )}{m{\omega }_{0}^{2}{q}_{{\rm{ref}}}},$$

with $$\bar{q}=q/{q}_{{\rm{ref}}}$$ and $${\bar{q}}^{{\prime} }={\rm{d}}\bar{q}/{\rm{d}}\tau$$.

The parameter values are obtained from the experiments and are listed in Table 1. For the numerical results, parameters of *Cantilever 1* are used. Besides that, the modal mass of the cantilever is calculated with the eigenfrequency and the modal stiffness using $m=k/{\omega }_{0}^{2}$. The continuation solver needs a periodic orbit as initial guess which is then continued using a pseudo-arclength continuation algorithm^17^ and continuation parameter *ζ*_s_. This initial orbit is calculated by ode45-solver in Matlab. During continuation the tip-sample distance at each computational step is used to calculate the corresponding value of *κ*_ts_. Additionally, the amplitude and phase of the cantilever are calculated for each solution orbit using an FFT algorithm. The corresponding amplitude and phase responsivities are computed with the gradient function in Matlab.

**Table 1:** Parameters used in experiment and computation

Parameter	Symbol	Cantilever 1	Cantilever 2
Eigenfrequency	*f*_0_ = *ω*_0_/(2*π*)	68 kHz	114 kHz
Modal Stiffness	*k*	38 N/m	178 N/m
Quality Factor	*Q* ≈ 1/(2*D*)	421	498
Tip Radius	*R*	27 nm	19 nm
Free Amplitude	*q*_0_	{1, 2, 5, 10} nm	
Hamaker Constant^20^	*A*	12 ⋅ 10^−20^ J	
Intermolecular Distance^21^	*a*_0_	0.4 nm	
Temperature	*T*	300 K	
Boltzmann Constant	*k*_B_	1.38 ⋅ 10^−23^ J/K	
Nondimen. Measurem. Bandwidth	*η*_B_	100	
Scaling Parameter Length	*q*_ref_	1 nm	

The simulations used for Fig. 2 are performed in Matlab Simulink using the same model as for the other simulations (see Eq. (25)) but for the parameters of *Cantilever 2* which is also used in the experiment. The significant difference is that this simulation does not calculate and continues steady-state solutions but is a time integration calculation. The tip-sample distance *ζ* is continuously reduced, resulting in an adjustable approach velocity *v*_a_ and a transient response of the cantilever. The amplitude of the cantilever is continuously evaluated by an implementation of a lock-in amplifier as used in the experiment. In that sense, this simulation acts as a virtual version of the described experimental setup with the benefit of being able to freely adjust the setup parameters.

### Experimental Amplitude-Distance Curves

For experimental validation of the derived model, it is necessary to measure amplitude-distance curves, also known as approach curves. This is realized using a self-built setup. The proposed setup can also be used for other research works, where commercial AFM systems are to restricted or not available.

For our measurements, we use so-called active cantilevers^18^ (active probes) with integrated electro-thermo-mechanical actuator and piezoresistive sensor from nanoanalytik, Ilmenau, Germany. For the measurements in Fig. 1b–d, *Cantilever 2* (parameters in Table 1) is used. The first bending eigenfrequency *f*_0_ and the quality factor *Q* are determined by a frequency sweep, *f*_0_ = 114172.61 Hz and *Q* = 498.49. The modal stiffness *k* of the first bending eigenmode is calculated by measuring 500 spectra of the cantilever’s thermal noise as described in Ref. ^19^, *k* = 178 N/m. The cantilever tip is made of silicon and has a radius of 19 nm as measured in a scanning electron microscope. The material of the sample is aluminum. Therefore, the Hamaker constant is assumed to be *A* = 12 ⋅ 10^−20^ J^20^ and the intermolucular distance *a*_0_ = 0.4 nm^21^. The measurement of the cantilever amplitude is done with a laser vibrometer OFV-5000 by Polytec, Waldbronn, Germany (displacement measurement resolution: 0.015 nm) and a lock-in amplifier Moku:Go by Liquid Instruments, San Diego, USA with filter cut-off frequency of 5 kHz. The piezo scanner PX 100 for approach of the sample to the cantilever is from piezosysteme jena, Jena, Germany. It is calibrated by measuring the applied voltage and the resulting displacement using a laser vibrometer, which provides an almost linear correlation between the applied voltage and displacement. The measurement of the various voltage signals is performed with several Handyscope HS5 oscilloscopes from TiePie engineering, Sneek, Netherlands (voltage measurement resolution: ≤4.8 mV). The entire measurement setup is placed under normal environmental conditions, which means that the temperature is 23^∘^C and the relative humidity is 41%.

In order to measure the process of the approach of the cantilever and the sample to each other, a precise and continuous movement of the piezo scanner needs to be achieved. Because the commercial controller belonging to the piezo scanner leads to insufficient movement, we developed a new analog electronic driver circuit, see Fig. 9. The idea is to treat the piezo scanner as a simple capacitance that can be either charged or discharged. Assuming a constant and noise-free voltage supply, the charging/discharging of the piezo scanner will lead to precise and continuous movement. The developed electronic circuit includes a half-bridge of MOSFET transistors and an IR2183 bridge driver for defined, overlap-free switching between the charging and discharging of the piezo scanner. The driver is controlled by an Arduino microcontroller that triggers the charging and discharging at previously defined amplitude thresholds of the cantilever, which are determined by the signal from the lock-in amplifier. The corresponding source code for the Arduino microcontroller can be found in the supplementary information files. During the approach, the voltage applied to the piezo scanner (*U*_Piezo_) is measured, as well as the lock-in amplifier signal and the trigger signal responsible for switching between the charging and discharging of the piezo scanner (sampling frequency 1 MHz). A short video showing the cantilever through the camera of the laser vibrometer when approaching the sample together with the measured lock-in amplifier signal can be found in the supplementary information files.

The post-processing of the measured data is as follows. First, the signal of the voltage applied to the piezo scanner is converted to a position signal by applying the calibration curve obtained as described before. Second, the relevant window of data corresponding directly to the actual approach is determined. Next, a straight line is fitted to the windowed position data, which is perfectly linear but contains high-frequency noise. This is acceptable because the dynamics of the piezo scanner do not allow following such high-frequency signals anyway (unloaded resonance frequency 790 Hz). Finally, the lock-in signal representing the amplitude of the cantilever (fully unfiltered) is plotted against the filtered position signal to obtain the presented amplitude-distance curves.

## Supplementary information

Video: AFM Approach

Source Code Arduino for Experimental Setup

## Data Availability

The raw data supporting the findings of this work is available from the corresponding author upon request.
